# Valeric acid attracts *C. elegans* by activating the AWC neurons through a *tax-4* -dependent signaling pathway

**DOI:** 10.17912/micropub.biology.001630

**Published:** 2025-05-24

**Authors:** Sreyan Sarkar, Lucero E. Rogel-Hernandez, Theresa Logan-Garbisch, Emily Fryer, Victoria Johnson, Miriam B. Goodman

**Affiliations:** 1 Molecular and Cellular Physiology, Stanford University, Stanford, California, United States; 2 Los Altos High School

## Abstract

Medicinal plants of the *Valeriana* genus have been traditionally used around the world to treat several nervous system disorders, yet our understanding of how they do so remains poorly understood. To deepen the understanding of their ability to influence nervous system pathways, we explored the ability of the model organism *Caenorhabditis elegans* to chemotax to crude extracts of *Valeriana officinalis* and found that *C. elegans* are weakly attracted to it. Upon investigating which chemical entities give rise to this behavior, we identified valeric acid (VA) as a primary candidate. Through chemotaxis assays, we show that wild-type *C. elegans* are strongly attracted to VA in a dose-dependent manner. Chemotaxis assays with mutant strains of *C. elegans* deficient in chemosensation indicate that the *tax-4* -dependent nervous pathways are most heavily responsible for detecting VA. However, *osm-9* -dependent pathways may also play a small role in regulating the worm's response to VA. Additionally, animals lacking AWC neurons are indifferent to this compound, and therefore, future research should focus on what molecular entities grant the AWC neurons the ability to detect VA.

**Figure 1. C. elegans is attracted to valerian extracts and valeric acid:**
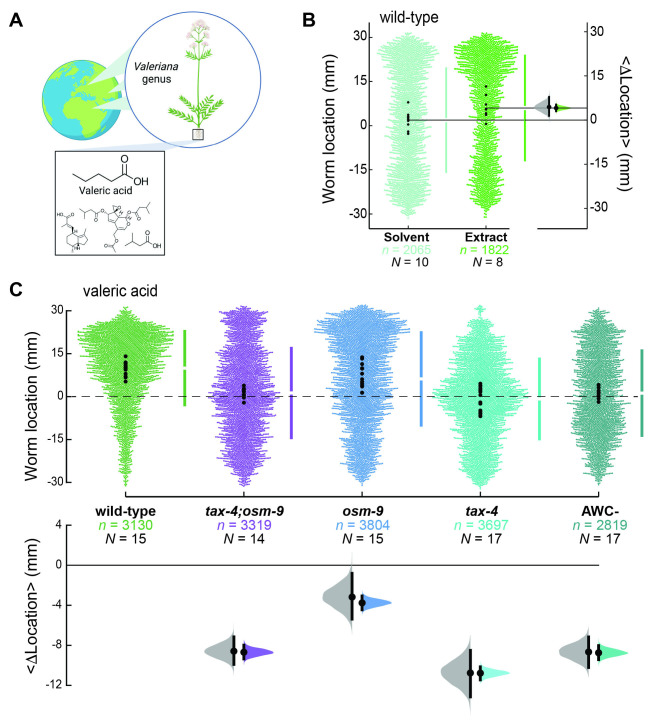
A) Illustration of the origin of *Valeriana* plants, highlighting regions in Europe, northern Africa, and the Middle East and selected compounds found in extracts of its roots: valeric acid, valerenic acid, valtrate, and isovaleric acid. Created in BioRender. Sarkar, S. (2025) https://BioRender.com/q3clzsa. B) Valerian root extracts attract wild-type *C. elegans* . Swarm plots (left axis) of the response to symmetric solvent (ethanol, left) and to ethanol extracts (right). Small dots in each swarm plot show the location of individual animals pooled ( *n* ) across *N* biological replicates and the large dots are the average position of each replicate. Vertical bars indicate the standard deviation and the gap between the bars shows the mean. Distribution of bootstrapped difference of the mean locations (right axis) derived from *N* replicates (gray) and from *n* animals pooled across replicates (green). 95% confidence intervals exclude zero in both cases. C) Valeric acid attracts *C. elegans* in a *tax-4* - and AWC-dependent manner. Swarm plots (top) showing the response to valeric acid as a function of the indicated genotype. Small dots in each swarm plot show the position of individual animals pooled ( *n* ) across *N* biological replicates, and the large dots are the average location of each replicate. To the right of each swarm plot, the gap indicates the mean worm location, and the bars represent the standard deviation. The bottom panel shows the bootstrapped difference of the mean locations compared to wild-type derived from *N* replicates (gray) and from *n* animals pooled across replicates (purple: *tax-4 ; osm-9* double mutant; blue: *osm-9* ; light aqua: *tax-4* ; dark aqua: AWC killed).

## Description

**Introduction**

Throughout history, humans have relied on plants to treat a wide range of conditions and to restore mental health (Wink, 2015). In parts of Europe, Northern Africa, and the Middle East, roots harvested from plants of the *Valeriana* genus (aka valerian) are traditionally used for treating anxiety, restlessness, sleep disorders, and epilepsy (Eadie, 2004; Patočka and Jakl, 2010; Shinjyo et al., 2020; Çelik and Kırmızıbekmez, 2025) ( Figure 1A ). The accredited therapeutic effects of valerian root are often attributed to the additive or synergistic effects of small molecules (SMs), compounds synthesized by plants to persist in their environment (Wink, 2015). Many of these plant-animal interactions reflect the ability of SMs to repel and attract animals, a behavior that relies upon chemical sensing by animals, by acting as ligands to key players of the nervous system.

*Caenorhabditis elegans* is a free-living nematode found in rotting fruits or vegetation (Frézal and Félix, 2015), an environment teeming with a rich array of chemicals, which may include SMs exuded by microbes and plant roots. Recently, we developed a high-throughput chemotaxis platform and used it to classify individual plant SMs as neutral, repellents, or attractants of *C. elegans* nematodes (Fryer et al., 2024). Chemotaxis behavior depends on subsets of 32 chemosensory neurons, which constitute ~10% of the entire *C. elegans* nervous system (Ferkey et al., 2021; Fryer et al., 2024). These chemosensory neurons convert SM ligand binding into electrical signals using ion channels, and all 32 chemosensory neurons express either the TAX-4 CNG channel or the OSM-9 TRPV channel. Adapting this platform to investigate a small number of chemical conductions, we determined whether crude ethanol extracts of valerian root ( *V. officinalis* ) are neutral, attractive, or repellent to wild-type *C. elegans* and to mutants lacking TAX-4 , OSM-9 , or both channels. Valerian extracts were weak attractants, which prompted us to investigate valeric acid (VA), one of the principal constituents of ethanol extracts of valerian (Shinjyo et al., 2020; Wu et al., 2023). In addition to being a component of valerian extract, VA seems to possess various health benefits, including anti-cancer, anti-diabetic, anti-inflammatory, and immunomodulatory, and interacts with molecular pathways in Alzheimer's disease, Parkinson's disease, and epilepsy (Kumari et al., 2024). VA attracts wild-type but not *tax-4* mutant *C. elegans .* These findings suggest that *C. elegans* may provide a useful platform for linking SMs to receptors in the nervous system.

**Methods**

**Worm husbandry:** We used standard techniques (Brenner, 1974) to grow *C. elegans* strains at 20°C on round 10 cm nematode growth medium plates seeded with *E. coli* OP50 . We age-synchronized our worms using hypochlorite treatment (Stiernagle, 2006) and gathered them as young adults for behavioral testing. We used five strains: N2 (Bristol), PR678 *tax-4 ( p678 )* III, CX10 *osm-9 ( ky10 )* IV, GN1077 *tax-4 ( p678 ) III; osm-9 ( ky10 )IV* and PY7502 *oyIs85 [ceh-36p::TU#813+ceh-36p::TU#814+srtx-1p::GFP+ unc-122p::DsRed]* . All strains are available from the Caenorhabditis Genetics Center, except for GN1077 , which was made in-house (Fryer et al., 2024) **.**

***Valeriana officinalis* growth and crude extract preparation:** We purchased *V. officinalis* seeds from J.L. Hudson, Seedsman (La Honda, CA). We planted seeds in a 24-cell tray filled with PRO-MIX and placed the tray inside a dark 4℃ incubator for 7 days to break seed dormancy. We moved the tray to a growth chamber at 25℃ and 45% humidity with a 12/12 hour photoperiod for 1 month. We selected individual plants and transplanted them into 3-gallon pots. We placed the plants back inside the growth chamber for 24 more months and watered them twice a month. We harvested their roots and macerated them in ethanol (0.98g/ml). We kept the maceration at room temperature for 3 days inside a dark drawer. We divided the resulting extract into aliquots (100 µl) and stored them at -20℃.

**Chemotaxis Assays:** We performed chemotaxis assays in four-well plates in which each lane held Gelrite® (gellan gum) and a foam insert defining the behavioral arena, as described in (Fryer et al., 2024). We spotted VA (2.5 μl; Sigma-Aldrich, CAS: 109-52-4) and solvent (2.5 µl; 70% ethanol) at opposite ends of the arena and waited 1 hour before depositing worms in the center. We used PVA eye spears (BVI, catalog no. NC0972725) saturated with valerian extract (40 μl) and ethanol (40 µl) to deliver the extract and solvent. Specifically, we placed treated eye spears on top of the foam insert next to the areas designated for compound and solvent placement. We washed worms twice with ddH _2_ O and once with chemotaxis buffer (5mM potassium phosphate, pH 6.0, 1mM MgSO _4_ , and 1mM CaCl _2_ ), then pipetted them onto the center of the arena. We delivered test chemicals and solvent at opposite ends of the assay arenas (±32.5mm from the center). Consult Fig. 1 of (Fryer et al., 2024) for a graphical representation of the assay arena. We placed assay plates inside a dry box (FORKSPARK® 30L, model: FSDCBLK30) or inside a cupboard. We conducted assays between 21.4 °C to 24.9 °C and 31% and 37% relative humidity. After 1 hour, we imaged assay plates with an Epson Perfection V600 Photo scanner (model: B11B198011) to acquire 1200 DPI/8-bit grayscale images, saved as *.tif files.

**Data curation and analysis:** We assigned each *.tif file and assay a unique tag. To annotate the worm locations in each assay, we used Our Worm Locator (OWL) (Fryer et al., 2024). The source code for the software is publicly available on GitHub . We included assays with at least 150 annotated worm locations in the analysis (Fryer et al., 2024). We analyzed the pooled data using estimation statistics (re-sampling 5000X) through the DABEST package (v. 0.3.1) (Ho et al. 2019). Additionally, we computed the mean worm location for each biological replicate and superimposed the values onto the pooled data to create a superplot and used estimation statistics to analyze these data as well. The data are visualized as swarmplots of worm location with the average location for each individual assay superimposed on the swarmplot. Source data and *.tif files are available at https://doi.org/10.25740/tb500hy5238 .

**Results**

Valerian extract is available commercially as tablets, powders, tinctures, or teas, and, in the US, these products are regulated as dietary supplements. As such, they are not always tested for manufacturing consistency and extract quality, or their origin may be uncertain. To study valerian extracts of known provenance, we grew valerian plants from seeds, harvested and macerated the roots, and prepared extracts using ethanol (see Methods). Wild-type *C. elegans* were weakly attracted to our custom valerian extract relative to the ethanol ( Figure 1B ). Searching the literature for SMs likely to be present in our extract (Shinjyo et al., 2020; Wu et al., 2023), we identified valeric acid (VA) as a promising lead.

Pure VA strongly attracts wild-type animals (Figure 1) and this was nearly 2-fold stronger than the response to a custom valerian extract: average locations following exposure to VA and valerian extract gradients were 9.95±12.86 mm (mean±s.d) and 5.98±17.80 mm (mean±s.d.), respectively. The stronger response to VA simplified further tests, including testing mutants carrying defects in the *tax-4* and *osm-9* ion channels for their response to VA. As found for dozens of other attractant chemicals (Fryer et al., 2024), *tax-4 ; osm-9* double mutants were indifferent to VA ( Figure 1C ). The *osm-9* gene is dispensable for this behavior, and *tax-4* is required ( Figure 1C ). Since the *tax-4* -expressing AWC neurons are linked to attraction to a variety of volatile chemicals ( *e.g.* , isoamyl alcohol, 2-heptanone, 2-butanone) of diverse structures (Ferkey et al., 2021), we investigated whether animals lacking these neurons can detect valeric acid (VA). Similar to ion channel mutants, animals lacking the AWC neurons are indifferent to VA. Collectively, these findings demonstrate that *C. elegans* are attracted to VA in a manner that depends on the *tax-4* CNG channel gene and the AWC neuron.

**Discussion**

Wild-type *C. elegans* are weakly attracted to valerian root extract and more strongly attracted to VA, one of several compounds commonly found in ethanol-based valerian root extracts. Like valerian itself, VA has potential as a therapeutic agent (Shi et al., 2021; Kumari et al., 2024). These findings suggest that future studies aimed at determining the chemical profile of valerian root extracts and the ability of these constituents to attract (or repel) *C. elegans* might be a fruitful strategy for gaining new insights into how chemicals produced by this plant affect behavior and nervous systems. Attraction to pure VA depends on the *tax-4* gene and the AWC neurons, which express both *tax-4* (Komatsu et al., 1996) and *osm-9* (Colbert et al., 1997). Loss of *osm-9* impairs attraction, suggesting that *osm-9* -dependent signaling also contributes to VA attraction. It is unclear if *tax-4* and *osm-9* both act in AWC to facilitate VA attraction. Given recent studies showing that many odorants and microbial metabolites activate multiple chemosensory neurons (Lin et al., 2023; Brissette et al., 2024), it seems likely that additional chemosensory neurons contribute to VA attraction. Both of these questions could be addressed using calcium imaging to determine how all chemosensory neurons, including AWC, respond to VA in wild-type, *tax-4* , and *osm-9* animals. Our finding that loss of the AWC neurons abrogates VA attraction strongly implies that AWC expresses a receptor for VA. Once identified, this receptor could help to identify candidate receptors for VA in humans.
